# Supplementary material for: Two distinct domains contribute to the substrate acyl chain length selectivity of plant acyl-ACP thioesterase
Source: Nat Commun. 2018 Feb 28;9:860. doi: 10.1038/s41467-018-03310-z (PMC5830452; doi:10.1038/s41467-018-03310-z)
Supplement: Supplementary file 1 — Supplementary Information [file 41467_2018_3310_MOESM1_ESM.pdf]

Supplementary Information

Two distinct domains contribute to the substrate acyl chain length selectivity of plant acyl-ACP thioesterase

Jing et al.

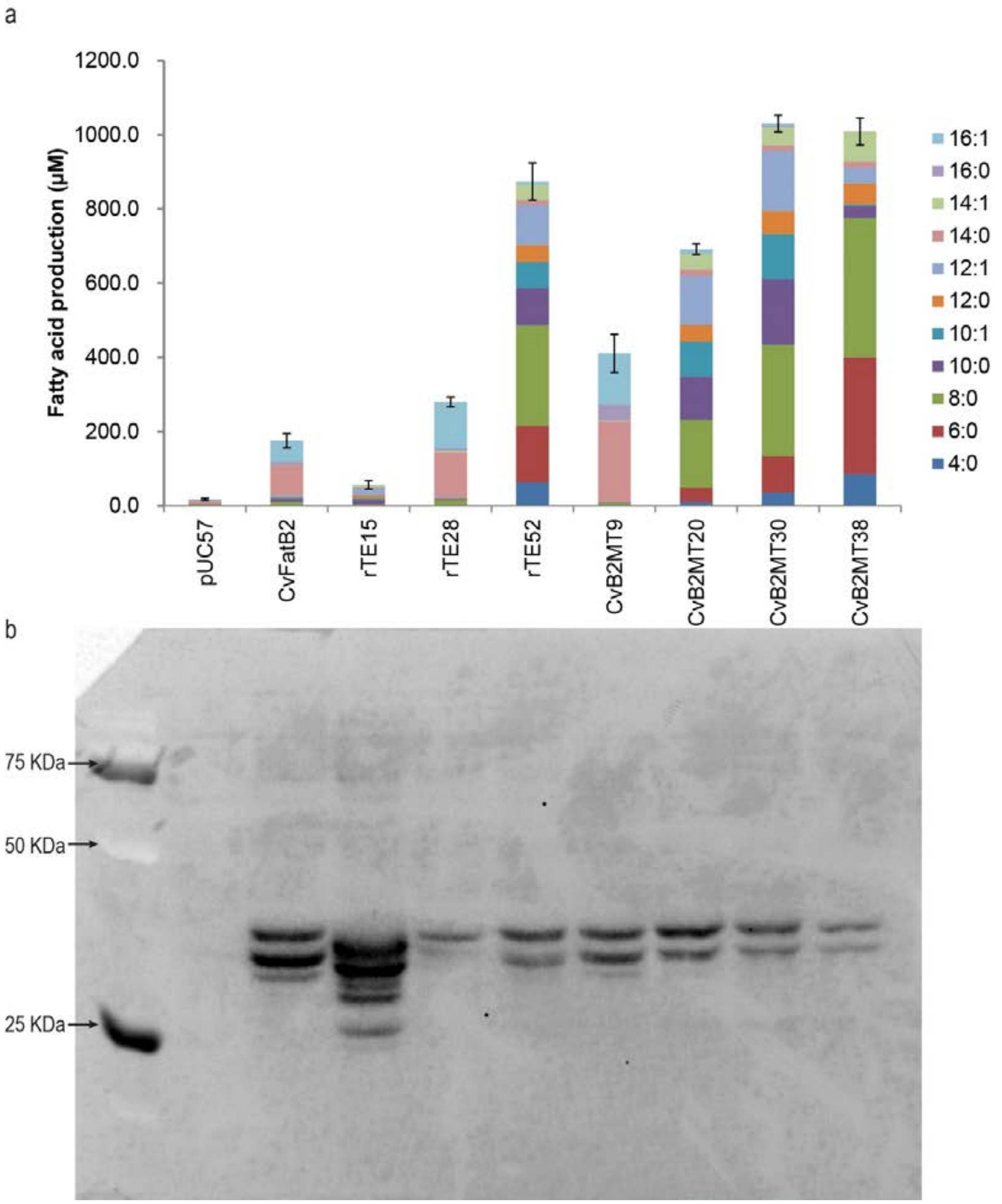

Supplementary Figure 1. Free FA titer and profile, and western blot analysis upon the expression of wild-type and seven representative CvFatB2 mutant enzymes in *E. coli* strains. Fatty acid titer is an average of 4 replicates, with error bars representing the standard error of the mean. The western blot analyses were conducted on 35 µg of soluble protein extracted from each *E. coli* strain, and CvFatB2 antibody was used to immunologically detect CvFatB2 variants. The multiple bands are probably the result of post-extraction proteolytic clipping of the expressed TEs. Because all the bands are immunologically recognized by our CvFatB2 antibody, we have integrated the signal from the entire collection of bands to compare the relative expression of the TE variants.

Supplementary Figure 2

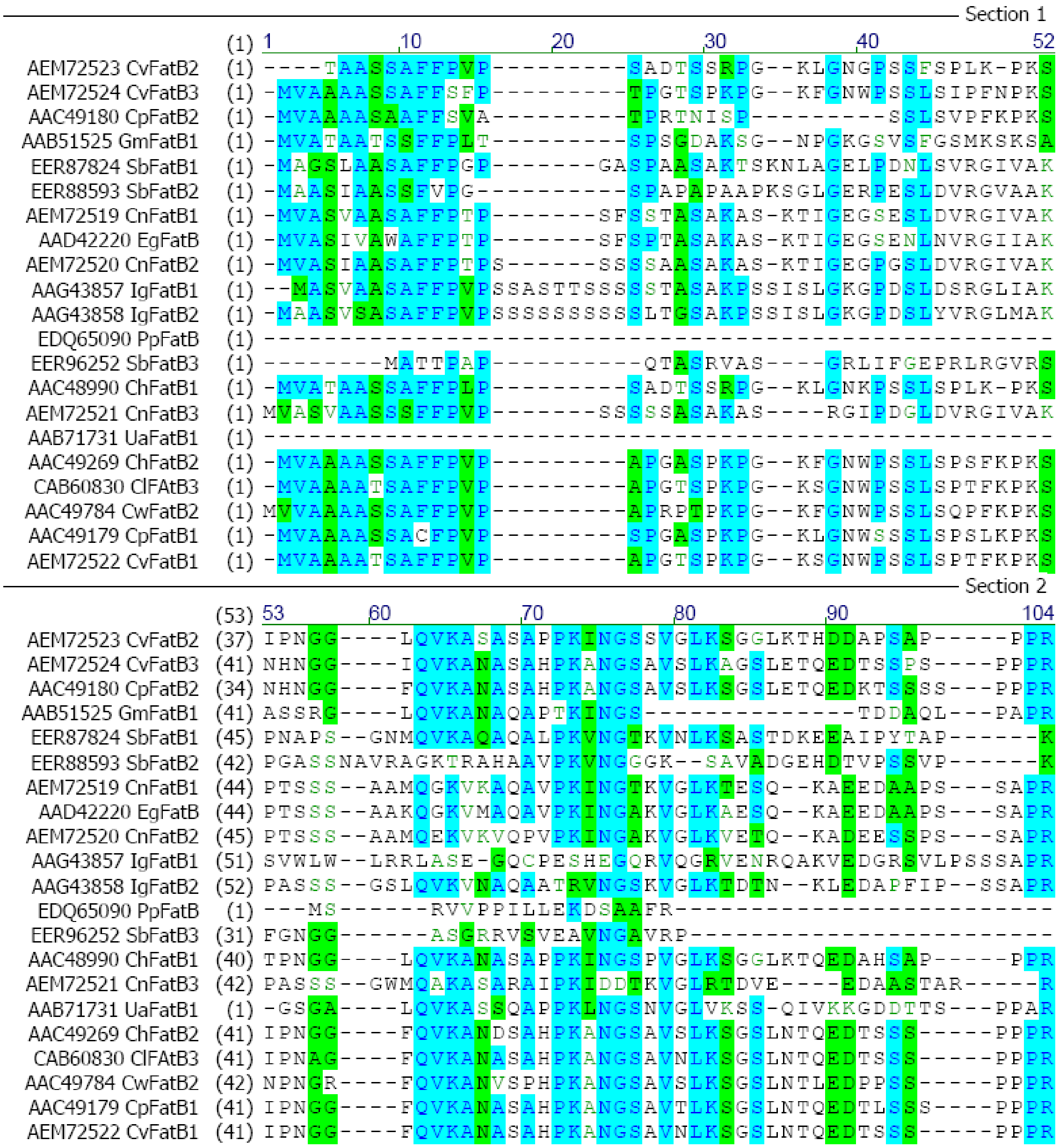

Supplementary Figure 2 continued

```
                                                                          Section 3
                  (105) 105  110              120             130             140             156
AEM72523 CvFatB2   (80) TFINQLPDWSMLLAAITTAFLAAEKQWMMLDRKPKRLDMLEDPFGLGRVVQD
AEM72524 CvFatB3   (85) TFISQLPDWSMLVSAITTVFVAAEKQWTMLDRKSKRPDVLVEPF-----VQD
AAC49180 CpFatB2   (79) TFINQLPVWSMLLSAVTTVFGVAEKQWPMLDRKSKRPDMLVEPLGVDRIVYD
AAB51525 GmFatB1   (72) TFINQLPDWSMLLAAITTVFLAAEKQWMMLDWKPRRPDMLIDTFGLGRIVQD
EER87824 SbFatB1   (89) TFYNQLPDWSMLLAAVTTIFLAAEKQWTLLDWKPKKPDMLVDTFGFGRIIQD
EER88593 SbFatB2   (86) TFYNQLPDWSMLLAAITTIFLAAEKQWTMLDWKPRRPDMLTDTFGFGRIIHD
AEM72519 CnFatB1   (89) TFYNQLPDWSVLLAAVTTIFLAAEKQWTLLDWKPRRPDMLTDAFSLGKIVQD
AAD42220 EgFatB    (89) TFYNQLPDWSVLLAAVTTIFLAAEKQWTLLDWKPRRPDMLTGAFSLGKIVQD
AEM72520 CnFatB2   (90) TFYNQLPDWSVLLAAVTTIFLAAEKQWTLLDWKPRRPDMLADAFGLGKIVQD
AAG43857 IgFatB1  (100) TFYNQLPDWSVLLAAITTIFLAAEKQWTLIDWKRGGPDMLTDAFGLGKIIEN
AAG43858 IgFatB2   (98) TFYNQLPDWSVLLAAITTIFLAAEKQWTLIDWKRGGPDMLSDAFGLPKIIEN
EDQ65090 PpFatB    (19) ----------AILAAIAGVALAAENQRRHDKT-----EVPVDVFRQGRLVES
EER96252 SbFatB3   (53) ----------NGAAAVADVRLVPAPPPASVEG-----DDGGDAFRLGKFVEG
AAC48990 ChFatB1   (83) TFINQLPDWSMLLAAITTVFLAAEKQWMMLDWKPKRPDMLVDPFGLGSIVQD
AEM72521 CnFatB3   (83) TSYNQLPDWSMLLAAIRTIFSAAEKQWTLLDSKKRGADAVADASGVGKMVKN
AAB71731 UaFatB1   (44) TFINQLPDWSMLLAAITTLFLAAEKQWMMLDWKPKRPDMLVDPFGLGRFVQD
AAC49269 ChFatB2   (84) TFLHQLPDWSRLLTAITTVFVK-SKRPDMHDRKSKRPDMLVDSFGLESTVQD
CAB60830 ClFAtB3   (84) AFLNQLPDWSMLLTAITTVFVAAEKQWTMLDRKSKRPDMLVDSVGLKSIVRD
AAC49784 CwFatB2   (85) TFLNQLPDWSRLRTAITTVFVAAEKQFTRLDRKSKRPDMLVDWFGSETIVQD
AAC49179 CpFatB1   (85) AFFNQLPDWSMLLTAITTVFVAPEKRWTMFDRKSKRPNMLMDSFGLERVVQD
AEM72522 CvFatB1   (84) AFLNQLPDWSMLLTAITTVFVAAEKQWTMLDRKSKRPDMLVDSVGLKSIVRD
                                                                          Section 4
                  (157) 157         170        180         190              208
AEM72523 CvFatB2  (132) GLVFRQNFSIRSYEIGADRTASIETVMNHLQETALNHVKTAGLSNDGFGRTP
AEM72524 CvFatB3  (132) GVSFRQSFSIRSYEIGVDRTASIETLMNIFQETSLNHCKSLGLLNDGFGRTP
AAC49180 CpFatB2  (131) GVSFRQSFSIRSYEIGADRTASIETLMNMFQETSLNHCKIIGLLNDGFGRTP
AAB51525 GmFatB1  (124) GLVFRQNFSIRSYEIGADRTASIETVMNHLQETALNHVKTAGLLGDGFGSTP
EER87824 SbFatB1  (141) GLVFRQNFLIRSYEIGADRTASIETLMNHLQETALNHVKTAGLLGDGFGATP
EER88593 SbFatB2  (138) GLMFRQNFSIRSYEIGADRTASIETLMNHLQETALNHVKTAGLLGDGFGSTP
AEM72519 CnFatB1  (141) GLIFRQNFSIRSYEIGADRTASIETLMNHLQETALNHVRNAGLLGDGFGATP
AAD42220 EgFatB   (141) GLVFRQNFSIRSYEIGADRTASIETLMNHLQETALNHVRNAGLLGDGFGATP
AEM72520 CnFatB2  (142) GLVFKQNFSIRSYEIGADRTASIETLMNHLQETALNHVKSAGLMGDGFGATP
AAG43857 IgFatB1  (152) GLIYRQNFSIRSYEIGADQTASIETLMNHLQETALNHVRCAGLLGNGFGSTP
AAG43858 IgFatB2  (150) GLLYRQKFSIRSYEIGADQTASIETLMNHLQETALNHVKCAGLLGNGFGSTP
EDQ65090 PpFatB    (56) RLVYGQTFVIRSYEIGADRTASIETMMNHFQETALNHVWMSGLAGDGFGATR
EER96252 SbFatB3   (90) TLVYRQQFVIRSYEIGPDRTATMETLMNLLQETALNHVMCSGLAGDGFGATL
AAC48990 ChFatB1  (135) GLVFRQNFSIRSYEIGADRTASIETVMNHLQETALNHVKIAGLSNDGFGRTP
AEM72521 CnFatB3  (135) GLVYRQNFSIRSYEIGVDKRASVEALMNHFQETSLNHCKCIGLMHGGFGCTP
AAB71731 UaFatB1   (96) GLVFRNNFSIRSYEIGADRTASIETLMNHLQETALNHVKSVGLLEDGLGSTR
AAC49269 ChFatB2  (135) GLVFRQSFSIRSYEIGTDRTASIETLMNHLQETSLNHCKSTGILLDGFGRTL
CAB60830 ClFAtB3  (136) GLVSRQSFLIRSYEIGADRTASIETLMNHLQETSINHCKSLGLLNDGFGRTP
AAC49784 CwFatB2  (137) GLVFRERFSIRSYEIGADRTASIETLMNHLQDTSLNHCKSVGLLNDGFGRTP
AAC49179 CpFatB1  (137) GLVFRQSFSIRSYEICADRTASIETVMNHVQETSLNQCKSIGLLDDGFGRSP
AEM72522 CvFatB1  (136) GLVSRHSFSIRSYEIGADRTASIETLMNHLQETTINHCKSLGLHNDGFGRTP
```

Supplementary Figure 2 continued

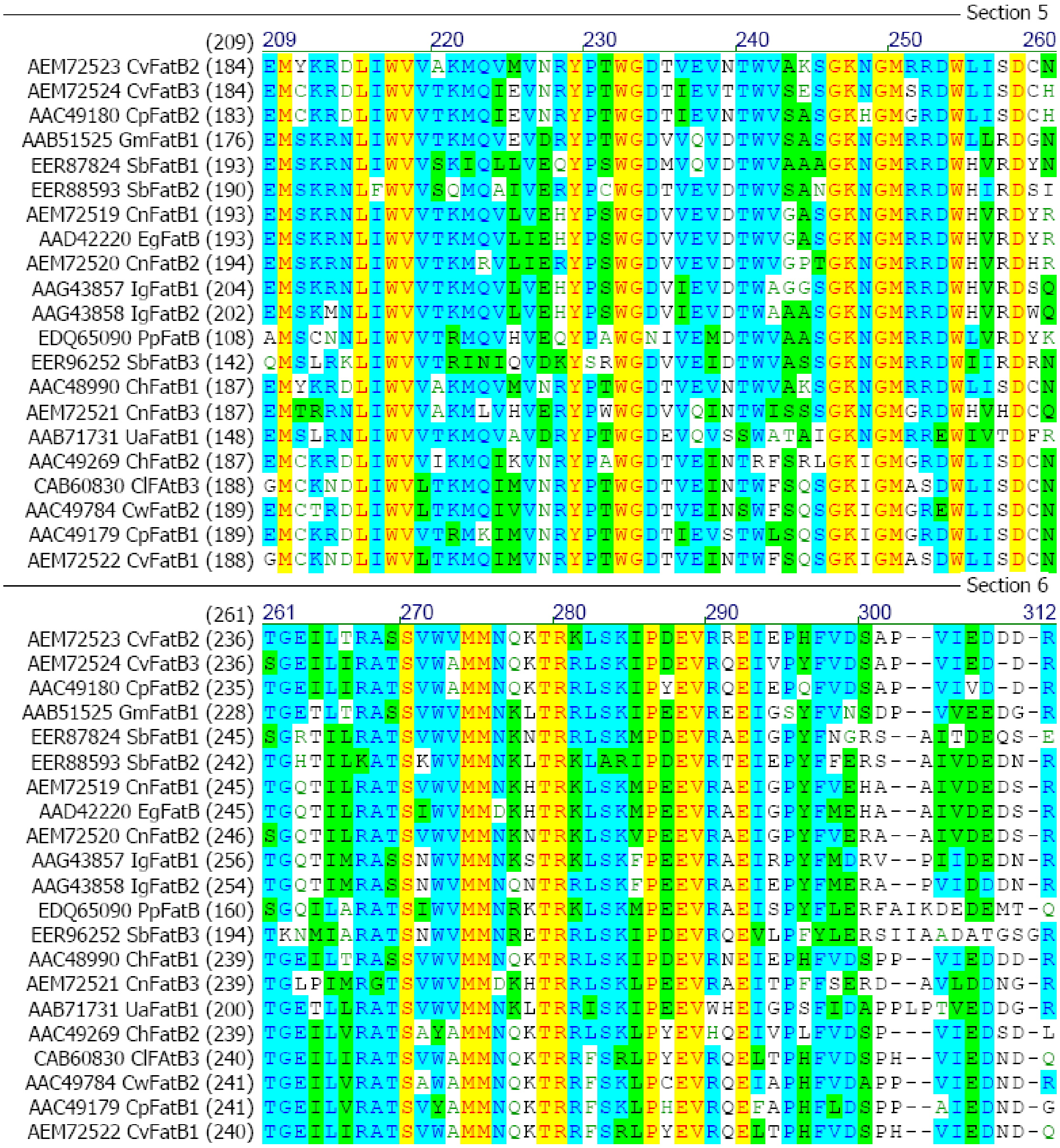

Supplementary Figure 2 continued

```
                                                                      Section 7
                        313       320       330       340       350          364
AEM72523 CvFatB2 (285)  KLPKLDEK-----SADSIRKGLTPRWNDLDVNQHVNNAKYIGWILESTPPEV
AEM72524 CvFatB3 (284)  KLHKLDVK-----TGDSIRNGLTPRWNDFDVNQHVNNVKYIAWLLKSVPTEV
AAC49180 CpFatB2 (283)  KFHKLDLK-----TGDSICNGLTPRWTDLDVNQHVNNVKYIGWILQSVPTEV
AAB51525 GmFatB1 (277)  KVTKLDDN-----TADFVRKGLTPKWNDLDINQHVNNVKYIGWILESAPQPI
EER87824 SbFatB1 (294)  KLAKPGSTSDGDTMKQFIRKGLTPRWGDLDVNQHVNNVKYIGWILESAPISI
EER88593 SbFatB2 (291)  KLPKLP-DGQSTSAAKYVRTGLTPRWADLDINQHVNNVKYIAWILESAPISI
AEM72519 CnFatB1 (294)  KLPKLD-----DDTADYIKWGLTPRWSDLDVNQHVNNVKYIGWILESAPISI
AAD42220 EgFatB (294)   KLPKLD-----DDTADYIKWGLTPRWSDLDVNQHVNNVKYIGWILESAPISI
AEM72520 CnFatB2 (295)  KLPKLD-----EDTTDYIKKGLTPRWGDLDVNQHVNNVKYIGWILESAPISI
AAG43857 IgFatB1 (305)  KLPKLD-----DDTADHVRSGLTPKWSDLDVNQHVNNVKYLGWILESAPISM
AAG43858 IgFatB2 (303)  KLPKLD-----DDTADHVRNGLTPRWSDLDVNQHVKNVKYIGWILESAPISI
EDQ65090 PpFatB (211)   KICRLNG------SAEYVRSGLTPRRSDLDMNQHVNNVKYIGWMLETVPPAV
EER96252 SbFatB3 (246)  KIEKLTDS-----TAEHIRSGLAPRWSDMDVNQHVNNVKYIGWILESVPLDV
AAC48990 ChFatB1 (288)  KLPKLDEK-----TADSIRKGLTPRWNDLDVNQHVNNVKYIGWILESTPPEV
AEM72521 CnFatB3 (288)  KLPKFD-----DDSAAHVRRGLTPRWHDFDVNQHVNNVKYVGWILESVPVWM
AAB71731 UaFatB1 (251)  KLTRFDES-----SADFIRXGLTPRWSDLDINQHVNNVKYIGWLLESAPPEI
AAC49269 ChFatB2 (287)  KVHKFKVK-----TGDSIQKGLTPGWNDLDVNQHVSNVKYIGWILESMPTEV
CAB60830 ClFAtB3 (289)  KLHKFDVK-----TGDSIRKGLTPRWNDLDVNQHVSNVKYIGWILESMPIEV
AAC49784 CwFatB2 (290)  KLHKFDVK-----TGDSICKGLTPGWNDFDVNQHVSNVKYIGWILESMPTEV
AAC49179 CpFatB1 (290)  KLQKFDVK-----TGDSIRKGLTPGWYDLDVNQHVSNVKYIGWILESMPTEV
AEM72522 CvFatB1 (289)  KLRKFDVK-----TGDSIRKGLTPRWNDLDVNQHVSNVKYIGWILESMPIEV

                                                                      Section 8
                        365  370       380       390       400             416
AEM72523 CvFatB2 (332)  LETQELCSLTLEYRRECGRESVLESLTAVDPSGEGYG--------------S
AEM72524 CvFatB3 (331)  FETQELCGLTLEYRRECRRDSVLESVTAMDPSKEGDR--------------S
AAC49180 CpFatB2 (330)  FETQELCGLTLEYRRECGRDSVLESVTAMDPSKEGDR--------------S
AAB51525 GmFatB1 (324)  LETRELSAVTLEYRRECGRDSVLRSLTAVSGGGVGDLGHA---------GNV
EER87824 SbFatB1 (346)  LEKHELASMTLDYRKECGRDSVLQSLTTVAGECVDGHAD----------STI
EER88593 SbFatB2 (342)  LENHELASIVLDYKRECGRDSVLQSHTSVQTDCNSESGE----------TTL
AEM72519 CnFatB1 (341)  LENHELASMTLEYRRECGRDSVLQSLTAISNDCTGGLPE----------ASI
AAD42220 EgFatB (341)   LENHELASMTLEYRRECGRDSVLQSLTAVANDCTGGLPE----------ASI
AEM72520 CnFatB2 (342)  LENHELASMSLEYRRECGRDSVLQSLTAVSNDLTDGLVE----------SGI
AAG43857 IgFatB1 (352)  LESHELASFTLEYRRECGRDGVLQSLTAVSADCSAGPAE----------LPI
AAG43858 IgFatB2 (350)  LESHELASMTLEYRRECGRDSVLQSLTSVSNNCTDGSEE----------LPI
EDQ65090 PpFatB (257)   LDGYELVSMNLEYRRECGQSDVVQSMT------------------TADGGNL
EER96252 SbFatB3 (293)  LEDYHLTSITLDYRRECRQSQLLESLTSMAMAMTSSPPAEPPLASSLCGSDL
AAC48990 ChFatB1 (335)  LETQELCSLTLEYRRECGRESVLESLTAMDPSGGGYG--------------S
AEM72521 CnFatB3 (335)  LDGYEVATMSLEYRRECRMDSVVQSLTAVSSDHADG-------------SPI
AAB71731 UaFatB1 (298)  HESHEIASLTLEYRRECGRDSVLNSATKVSDSSQLGKS------------AV
AAC49269 ChFatB2 (334)  LETQELCSLALEYRRECGRDSVLESVTAMDPSKVGVR--------------S
CAB60830 ClFAtB3 (336)  LETQELCSLTVEYRRECGMDSVLESVTAVDPSENGGR--------------S
AAC49784 CwFatB2 (337)  LETQELCSLTLEYRRECGRESVVESVTSMNPSKVGDR--------------S
AAC49179 CpFatB1 (337)  LETQELCSLTLEYRRECGRDSVLESVTSMDPSKVGDR--------------F
AEM72522 CvFatB1 (336)  LETQELCSLTVEYRRECGMDSVLESVTAVDPSENGGR--------------S
```

Supplementary Figure 2 continued

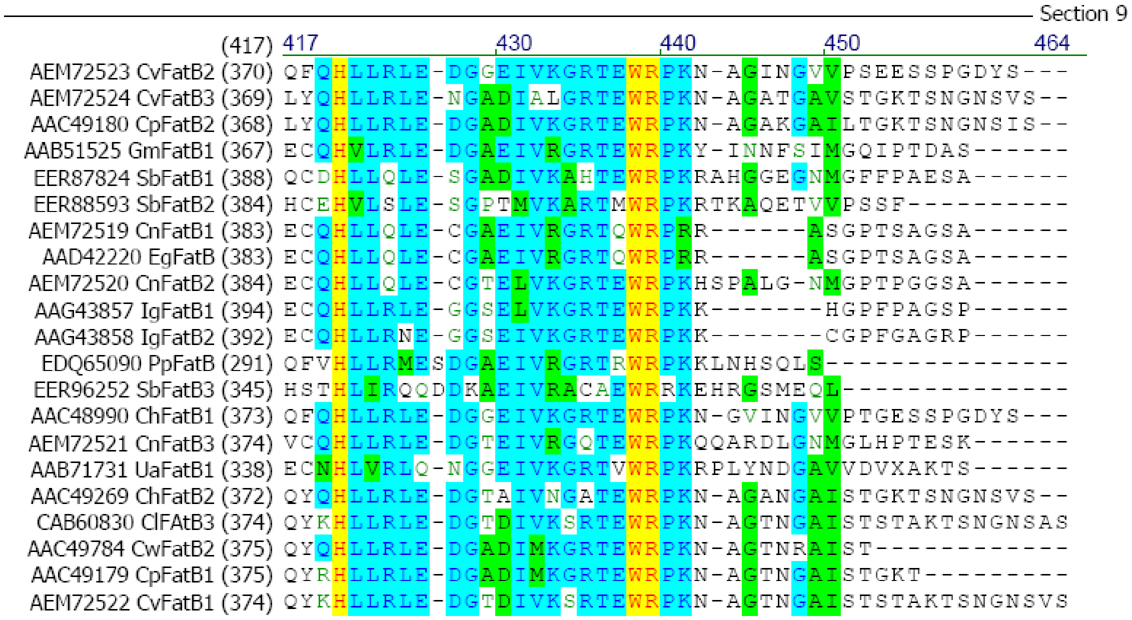

Supplementary Figure 2. Multiple sequence alignment of 21 functionally characterized plant acyl-ACP TEs. The alignment was constructed with Vector NTI - Alignment X, and the default settings identify identical residues (yellow highlighted), highly conserved residues (blue highlighted), weakly similar residues (green highlighted), and non-similar residues are not highlighted. Sequences are identified by their GenBank Accession Numbers, and source organism: Cv, *Cuphea viscosissima*; Cp, *Cuphea palustris*; Gm, *Garcinia mangostana*; Sb, *Sorghum bicolor*; Cn, *Cocos nucifera*; Eg, *Elaeis guineensis*; Ig, *Iris germanica*; Pp, *Physcomitrella patens*; Ch, *Cuphea hookeriana*; Ua, *Ulmus americana*; Cl, *Cuphea lanceolata*; Cw, *Cuphea wrightii*.

Supplementary Table 1. DNA primers used for the amplification of six overlapping fragments of CvFatB1 and CvFatB2 used in domain-swapping experiments

Domain-swapping fragment	Forward primers	Reverse primers
I	pUC57F (5'-CGGCTCGTATGTTGTGTGGAAT-3')	Cv-R2 (5'-GGTACGATCCGCGCCGATTTC-3')
II	Cv-F2 (5'-GAAATCGGCGCGGATCGTACC-3')	Cv-R3 (5'-CCAGGTCGGATAACGATTGAC-3')
III	Cv-F3 (5'-GTCAATCGTTATCCGACCTGG-3')	Cv-R4 (5'-CGGGTTTTCTGGTTCATCAT-3')
IV	Cv-F4 (5'-ATGATGAACCAGAAAACCCG-3')	Cv-R5 (5'-TCGTTCCAACGCGGCGTCAGACC-3')
V	Cv-F5 (5'-GGTCTGACGCCGCGTTGGAACGA-3')	Cv-R6 (5'-ATCTTCCAGACGCAGCAG-3')
VI	Cv-F6 (5'-CTGCTGCGTCTGGAAGAT-3')	pUC57R (5'-CTGCAAGGCGATTAAGTTGGGTAAC-3')

Supplementary Table 2. Comparison of the 15 residues that are predicted to affect substrate specificity of acyl-ACP TEs

	**Fragments**	II	III								IV					
	**Residue Number**	194[a]	213	217	218	219	223	226	227	248	257	260	268	276	282	289
**Class I [b]**	AEM72523 CvFatB2	V	V	V	A	K	N	R	R	V	L	I	I	A	D	L
	AEM72524 CvFatB3	V	V	V	S	E	N	S	R	A	L	I	I	A	D	L
	AAC49180 CpFatB2	V	V	V	S	A	H	G	R	A	L	I	I	A	D	L
	AAB51525 GmFatB1	V	V	V	S	A	N	R	R	V	L	I	I	D	D	L
	EER87824 SbFatB1	V	V	V	A	A	N	R	R	V	L	M	I	S	Q	P
	EER88593 SbFatB2	V	V	V	S	A	N	R	R	V	L	I	I	S	D	L
	AEM72519 CnFatB1	V	V	V	G	A	N	R	R	V	L	M	I	A	D	L
	AAD42220 EgFatB	V	V	V	G	A	N	R	R	V	L	M	I	A	D	L
	AEM72520 CnFatB2	V	V	V	G	P	N	R	R	V	L	V	I	A	D	L
	AAG43857 IgFatB1	V	V	A	G	G	N	R	R	V	L	F	I	V	D	L
	AAG43858 IgFatB2	V	V	A	A	A	N	R	R	V	L	F	I	A	D	L
	EDQ65090 PpFatB	V	M	V	A	A	N	R	R	V	L	M	I	F	M	L
	EER96252 SbFatB3	V	I	V	A	S	N	R	R	V	L	I	V	S	S	L
	AAC48990 ChFatB1	V	V	V	A	K	N	R	R	V	L	I	I	P	D	L
**Class II and III**	AEM72521 CnFatB3	V	I	I	S	S	N	G	R	V	L	L	I	D	N	F
	AAB71731 UaFatB1	V	V	A	T	A	N	R	R	V	I	I	I	P	D	F
	AAC49269 ChFatB2	V	I	F	S	R	I	G	R	A	L	L	I	P	S	F
	CAB60830 ClFatB3	L	I	F	S	Q	I	A	S	A	F	L	L	P	N	F
	AAC49784 CwFatB2	L	I	F	S	Q	I	G	R	A	F	L	I	P	N	F
	AAC49179 CpFatB1	V	V	L	S	Q	I	G	R	A	F	L	F	P	N	F
	AEM72522 CvFatB1	L	I	F	S	Q	I	A	S	A	F	L	L	P	N	F

a. Numbering of the residues are according to the sequence of CvFatB2 (GenBank Accession AEM72523.1), and their positions among the domain-swapping fragments are indicated; b. Each acyl-ACP TE is identified as either belonging to the FatB subclass I or II and III [1], and they are each identified by their GenBank Accession Numbers, and source organism: Cv, *Cuphea viscosissima*; Cp, *Cuphea palustris*; Gm, *Garcinia mangostana*; Sb, *Sorghum bicolor*; Cn, *Cocos nucifera*; Eg, *Elaeis guineensis*; Ig, *Iris germanica*; Pp, *Physcomitrella patens*; Ch, *Cuphea hookeriana*; Ua, *Ulmus americana*; Cl, *Cuphea lanceolata*; Cw, *Cuphea wrightii*.

Supplementary Table 3. FA composition and total free FA accumulation of *E. coli* strains expressing acyl-ACP TE mutants

Sample name	mol percentage (%)											Total free FAs (μM)
	4:0	6:0	8:0	10:0	10:1	12:0	12:1	14:0	14:1	16:0	16:1	
CvB2MT17	2.0	1.7	26.2	6.5	1.5	17.6	13.5	8.6	15.8	5.0	1.6	114±2
CvB2MT6	4.6	3.1	38.2	5.8	0.2	21.7	9.1	3.4	13.9	0.0	0.0	31±1
CvB2MT34	6.1	3.9	31.8	9.6	6.3	9.2	18.4	2.8	11.2	0.3	0.4	464±6
CvB2MT33	5.8	2.5	27.2	7.9	5.3	9.6	20.4	5.1	12.5	0.0	3.6	570±4
CvB2MT25	4.5	3.6	28.8	9.3	3.4	13.0	15.9	5.2	13.7	2.0	0.6	285±6
CvB2MT47	0.6	0.9	18.5	12.0	4.2	17.9	17.5	1.4	4.5	22.6	0.0	192±14
CvB2MT45	1.9	2.0	22.5	15.8	6.2	13.1	24.4	2.6	11.4	0.0	0.0	574±37
CvB2MT44	0.7	3.5	22.7	23.6	11.3	7.9	24.2	1.8	4.0	0.0	0.2	713±34
CvB2MT42	1.8	7.1	27.0	15.0	12.1	6.9	18.9	2.6	6.1	1.5	1.1	823±11
CvB2MT20	1.5	5.6	26.2	16.9	13.6	6.6	19.1	2.4	6.2	0.3	1.4	691±14
CvB2MT30	3.5	9.5	29.2	17.2	11.7	6.1	15.7	1.5	4.7	0.4	0.6	1031±22
CvB2MT48	0.1	1.2	13.7	38.6	18.1	6.7	17.5	0.0	3.9	0.2	0.0	137±4
CvB2MT40	1.8	4.8	61.4	12.5	8.5	3.3	6.2	0.6	0.9	0.0	0.0	1068±57
CvFatB1	0.0	3.5	45.3	26.1	8.9	3.9	9.6	1.1	0.9	0.0	0.6	542±32
CvB2MT29	2.4	1.5	11.4	7.2	2.5	15.0	18.1	12.1	20.5	0.4	8.9	287±12
CvB2MT12	2.2	1.6	12.5	7.2	1.6	16.4	15.4	15.3	20.0	0.0	7.8	149±4
CvB2MT2	1.0	0.7	8.9	4.6	1.1	10.0	9.7	24.5	19.9	0.1	19.5	82±8
CvB2MT32	3.1	1.3	21.5	7.3	3.9	4.8	14.4	9.5	30.6	0.0	3.4	331±11
CvB2MT16	2.2	0.9	15.4	5.6	1.6	3.9	9.6	22.8	30.9	0.0	7.0	308±19
CvB2MT31	3.4	2.2	17.9	5.9	3.7	9.0	19.6	12.1	17.3	0.7	8.1	393±7
CvB2MT19	4.4	2.1	18.2	6.3	2.8	10.3	22.0	8.5	18.4	0.6	6.3	168±6
CvB2MT15	4.0	2.1	16.1	7.5	1.2	15.6	19.8	9.2	19.8	0.7	4.0	150±6
CvB2MT27	3.3	2.0	20.8	9.9	5.8	10.3	21.5	5.3	17.6	0.3	3.2	230±5
CvB2MT18	4.2	1.9	21.9	8.4	3.1	13.6	19.9	6.1	15.3	2.3	3.2	300±8
CvB2MT22	2.9	1.6	20.7	9.2	1.5	13.0	15.6	8.9	16.7	0.0	10.0	85±6
CvB2MT10	3.0	2.2	21.6	9.6	4.6	8.8	14.1	9.8	16.4	0.0	9.9	171±13
CvB2MT38	8.5	31.1	37.2	3.1	0.6	5.7	4.2	1.6	8.1	0.0	0.0	1009±36
CvB2MT35	10.5	23.6	43.1	2.5	0.6	3.6	4.1	3.0	8.8	0.0	0.1	124±4
CvB2MT41	15.1	23.9	36.2	3.8	18.9	1.4	0.7	0.0	0.0	0.0	0.0	93±7
CvB2MT26	6.3	17.2	31.7	1.2	0.0	4.8	0.0	17.9	3.9	11.3	5.7	41±3
CvB2MT36	22.1	35.2	40.3	0.0	0.0	0.0	0.0	2.4	0.0	0.0	0.0	46±2
CvB2MT1	19.4	46.0	34.6	0.0	0.0	0.0	0.0	0.0	0.0	0.0	0.0	2.4±0.8
CvB2MT37	8.9	40.1	40.2	3.4	0.3	2.8	1.0	1.1	2.0	0.0	0.2	333±12

CvB2MT43	0.4	0.7	4.1	1.2	0.5	1.5	1.4	47.0	0.7	11.5	31.0	174±11
CvB2MT9	0.1	0.1	1.5	0.3	0.1	0.3	0.4	52.6	0.6	10.4	33.7	411±51
CvB2MT14	0.0	0.4	3.4	0.8	0.0	1.6	0.8	52.2	0.7	14.1	26.0	108±1
CvB2MT11	0.1	0.4	4.3	1.5	0.2	3.8	4.0	45.5	4.9	11.9	23.5	120±8
CvB2MT13	1.0	0.8	12.7	2.1	0.7	0.9	1.5	50.7	0.5	3.1	26.0	183±6
CvFatB2	0.0	0.3	5.4	5.4	1.4	1.3	3.2	45.2	0.6	5.5	31.7	175±19
CvB2MT23	0.6	0.4	5.6	1.0	0.3	0.8	0.8	54.2	0.9	6.6	28.7	385±16
CvB2MT8	0.2	0.4	3.9	0.3	0.0	0.0	0.0	58.0	0.0	4.4	32.8	74±6
CvB2MT7	0.7	0.7	6.1	0.6	0.0	0.0	0.0	62.2	0.0	0.9	28.8	47±13
CvB2MT4	0.2	0.3	3.7	0.6	0.1	0.0	0.2	60.9	0.0	9.3	24.5	199±18
CvB2MT24	0.2	0.1	3.6	0.4	0.1	0.0	0.3	73.6	0.2	1.4	20.0	401±49
CvB2MT5	0.0	0.1	1.5	0.2	0.0	0.0	0.0	81.5	0.0	2.9	13.9	457±33
CvB2MT28	1.7	1.4	17.6	5.0	1.2	7.5	3.7	40.1	4.1	3.4	14.3	328±21
CvB2MT21	1.9	1.1	17.8	3.4	0.6	4.6	2.7	43.8	4.5	2.0	17.5	201±7
CvB2MT3	2.6	1.9	17.2	4.1	1.5	2.1	4.6	40.1	4.3	0.0	21.6	90±6

Green-shading identifies the major fatty acids produced by each strain expressing the mutant acyl-ACP TE enzyme.

Supplementary Reference

1. Jing, F. et al. Phylogenetic and experimental characterization of an acyl-ACP thioesterase family reveals significant diversity in enzymatic specificity and activity. *Bmc Biochem* **12**, 44 (2011).
