# Supplementary material for: Two distinct domains contribute to the substrate acyl chain length selectivity of plant acyl-ACP thioesterase
Source: Nat Commun. 2018 Feb 28;9:860. doi: 10.1038/s41467-018-03310-z (PMC5830452; doi:10.1038/s41467-018-03310-z)
Supplement: Supplementary file 3 — Description of Additional Supplementary Files [file 41467_2018_3310_MOESM3_ESM.pdf]

Description of Additional Supplementary Files

File Name: Supplementary Movie 1
Description: A rotary view of the acyl-binding cavity in the N-terminal hotdog domain of 2OWN. The residues that form the cavity are shown as magenta and yellow stick models. The four bulky residues that occupy more than 50% of the surface area of the acyl-binding cavity are shown as yellow stick models. Catalytic residues are shown as red stick models. The surface is shown in 20% transparency.
